# Impact of the Consistency of Food Substances on Health and Related Factors of Residents in Welfare Facilities for Seniors in Japan

**DOI:** 10.3390/dj8010009

**Published:** 2020-01-14

**Authors:** Reiko Sakashita, Takuichi Sato, Hiroshi Ono, Akiko Hamaue, Misao Hamada

**Affiliations:** 1 College of Nursing Art and Science, University of Hyogo, Akashi 673-8588, Japan; hiroshi_ono@cnas.u-hyogo.ac.jp (H.O.); akiko_hamaue@cnas.u-hyogo.ac.jp (A.H.); 2 Department of Medical Technology, Niigata University Graduate School of Health Sciences, Niigata 951-8518, Japan; tak@clg.niigata-u.ac.jp; 3 Social Welfare Corporation Lavita, Osaka 554-0002, Japan

**Keywords:** food consistency, oral function, number of teeth, calorie intake, welfare facility

## Abstract

The aim of this study is to determine the effect of food consistency on health and related factors among residents in welfare facilities for seniors (n = 227; mean age, 86.2 ± 8.0 years; 78.9% female). Residents who ate regular food had a lower incidence of fever during the 3-month period (*p* < 0.001) and consumed more calories (1325.97 ± 220.2 kcal) than those who ate chopped (1125.0 ± 256.8 kcal), paste (1122.0 ± 288.5 kcal), and gastric tube food (812.5 ± 150.7 kcal) (*p* < 0.001). Modifying a resident’s food by making it softer and finer did not reduce the incidence of choking. Logistic regression analysis (backward elimination method) revealed four factors related to eating regular food: vitality index, appetite, number of remaining teeth, and choking frequency. Causal relationships were not obtained because this was a cross-sectional study. The findings of this study suggest that a regular consistency of food positively influences the health of older individuals.

## 1. Introduction

Pneumonia is the leading cause of death in older individuals in many countries, including Japan [1,2]. In particular, aspiration is an important mechanism contributing to the pathogenesis of pneumonia in older patients, and its management is a major medical problem [3]. The risk factors for aspiration pneumonia included poor oral function, difficulties in swallowing and poor oral hygiene, as well as the presence of a nasogastric tube, dementia, stroke, use of sedatives, and agitation.

When an elderly individual has a problem in mastication or swallowing, the textures of their food are modified according to his/her capability [4,5] by altering the foods’ consistency from a regular to a “modified” texture. Currently, however, there is no international consensus on the terminology and definitions of different consistencies of foods and fluids because of the variations across countries [6]. For instance, the International Dysphagia Diet Standardisation Initiative (IDDSI) has standardized five levels of dysphagia diet terminology, ranging from a liquidized to regular texture [6]. In contrast, Japan’s Ministry of Health Labor and Welfare (JMHW) has defined six levels as follows—level 0: smooth jelly foods without protein, level 1: smooth jelly foods with protein, level 2: jelly foods with protein, level 3: paste containing meat/fish, level 4: soft foods, and level 5: normal diet [7]. Despite these definitions, chopped and pasty foods are generally used in Japan during admission to the hospital or nursing facilities, often becoming to use gastric tubes.

Chewing of regular foods is expected to result in the following benefits: (1) preserving and promoting relevant muscle function; (2) promoting secretion of saliva, which contains immune substances such as immunoglobulin A (IgA) [8,9], and has buffering effects that prevent increased acidification (dental caries) [8,9], (3) moistening effects by saliva, and thus promoting the functions of chewing, swallowing, mucosal protection, and denture adsorption [8,9], and (4) food digestion and absorption mediated by digestive enzymes [8,9].

Programs to improve the meals of residents and aid in their health recovery have been reported; these programs are generally focused on the transition from gastric tube feeding to oral feeding or nutritional supplementation [10,11]. Further efforts are needed to enable senior residents to eat foods with consistencies close to those of regular foods. Additionally, the relationship between regular food ingestion and actual health conditions has not been sufficiently clarified. Therefore, in this study, the relationship between the physical properties of foods, such as consistencies, and the health conditions of older people living in welfare facilities, was investigated.

This study aims to focus on the following four points:

(a) Is the consistency of food related to the incidence of pneumonia and fever?
(b) Is the consistency of food related to calorie intake and nutritional conditions?
(c) Is the consistency of food related to choking?
(d) What are the factors that enable older people to eat regular food?

## 2. Methods

### 2.1. Participants

The subjects were elderly people living in welfare facilities in Japan (n = 227; mean age, 86.2 ± 8.0 years; male 48 and female 179; Table 1). The target population was elderly people over 60 living in welfare facilities in Japan. A non probability convenient sampling technique was used to select facilities, including all elderly people living in the data collection site, who met the inclusion criteria and accepted participation in the research. Data obtained from the three welfare facilities were integrated for analysis due to the limited marked differences in subject composition and research results.

### 2.2. Research Items

#### 2.2.1. Consistencies of Food

The consistencies of food were categorized into five levels, and the data were collected from the records kept by the welfare facilities: regular food—soft-solid food (prepared by boiling regular food until it becomes soft or by cutting them into bite-size pieces), chopped food (prepared by chopping regular food into small pieces, ca. 5–10 mm in length), paste food (prepared by mixing foodstuff with water until the mixture turns into paste), and food for those who had percutaneous endoscopic gastrostomy (for gastric tube feeding).

#### 2.2.2. General Health Conditions

For the following six items (a–f), the relevant data were collected from the records maintained by the welfare facilities, while the three remaining items (g–i) were obtained by asking the staff in-charge.

(a) Level of care needed: According to the standards established by JMHW, the extent of nursing care needs was classified into five levels, with 1 being the lightest and 5 being the heaviest [7].
(b) Daily life degree of autonomy of older individuals with dementia: According to the standards established by JMHW and based on the severity of dementia, the degree of independence of older individuals is classified into seven levels, from the self-reliant level to the level accompanied by serious physical symptoms.
(c) Past history: Information regarding past history was obtained from the records in the nursing care stations.
(d) Past history of pneumonia or fever: The records were checked for past history of pneumonia and presence/absence of episodes of fever (body temperature exceeding 37.5 °C or ≥1 °C increase of an individual’s average body temperature for a week) within three months before the initiation of research, although the possibility of the cause of the fevers by other than pneumonia (such as flu and other infections) cannot be excluded based on merely the past episodes of fever.
(e) Body mass index (BMI): The participant’s height (m) and body weight (kg) were used to calculate the BMI ([body weight]/[height]^2^) as the index of nutritional status.
(f) Calorie and water intake: A participant’s daily calorie intake was calculated by multiplying the daily calorie content of meals by the rate of intake (reflected in the weight), and the mean daily calorie intake for three days before the study was obtained. A participant’s daily water intake was obtained from the records of the nursing station, and the mean daily water intake for 3 days before the study was calculated.
(g) Appetite: The subjects were enquired about their appetite and instructed to select one of the following four options: “good appetite”, “moderate appetite”, “no appetite”, and “uneven appetite”. Subsequently, two categories of “no appetite” and “uneven appetite” were integrated into one category.
(h) Choking: The subjects were enquired about choking episodes and instructed to select one of the following three options: “few choking episodes”, “approximately 1–2 episodes daily”, and “choking episodes at every meal”.
(i) Vitality Index (VI): VI was developed as a scale to assess the quality of life of older people with dementia. They were enquired about their vitality on the following five aspects: upon waking up, communication, eating, excretion, and rehabilitation/activities. Vitality was expressed on a scale of 0–10, and a higher score indicates a higher level of willingness. It has been previously demonstrated that VI can evaluate willingness—regarded as a significant factor related to the activities of daily living (ADL) and levels of care needed—and determine the prognosis in life [12].

#### 2.2.3. Examination of the Oral Conditions

A dentist examined the oral cavity of the participants in the following manner with careful consideration of the lighting. The dentist evaluated the oral cavity for abnormal oral mucosa, dental caries (loss of tooth resulting from tooth decalcification), number of teeth and dentures, and a missing tooth was assessed if neither a natural tooth nor an artificial tooth was found in the respective position. The dentist also evaluated the occlusal conditions: good, if some natural or artificial teeth that could bite were present (Eichner’s classification group A [13]) or faulty (bad), and if only a few teeth that could bite were present because of loss of multiple teeth or aggravation of dental caries (modified from Eichner’s classification groups B and C [14]). A simplified tongue function test, whose reliability and validity have been confirmed [15], was conducted to examine the ability to extend and control the tongue. The ability to extend (tongue movement) was rated by asking the subject to stick out his/her tongue beyond the front line of the lower lip, which was observed and rated as follows: 0 (cannot move the tongue), 1 (cannot extend the tongue beyond the frontline of the lower lip), 2 (can extend the tongue beyond the frontline of the lower lip by <1 cm), and 3 (can extend the tongue beyond the frontline of the lower lip by >1 cm); while tongue control was rated as follows: 0 (cannot move the tongue), 1 (cannot stick out the tongue straight and maintain it), and 2 (can stick out the tongue straight and maintain it).

### 2.3. Data Analysis Methods

Descriptive statistical methods were used to test each variable. Continuous variables were presented as mean ± standard deviation. The χ^2^ test was used to evaluate the relationship between the consistency of food and the categorical variables. One-way analysis of variance was used to evaluate the relationship between the consistency of food and the variables that might contribute parametrically. If any significant difference was observed, the Tukey–Kramer method was used to perform multiple comparisons.

The associations between regular food intake (whether the subject ate regular food or not) and the age, level of care needed, daily life degree of autonomy, past history of pneumonia, presence of fever, frequency of choking, appetite, VI, number of teeth, occlusal conditions, tongue extension, and tongue control were analyzed with univariate logistic regression analysis. Next, all variables which showed a significant relationship (*p* < 0.05) were tested simultaneously in a multiple logistic regression analysis with stepwise backward elimination to determine useful predictors. Before multiple regression analysis, we ruled out relevant correlations of any of the predictors with each other (Spearman’s *r* < 0.5) to solve multicollinearity. Statistical analysis was performed using SPSS PASW Statistics v17 (SPSS Inc., Chicago, IL, USA).

### 2.4. Ethical Considerations

The study was conducted in accordance with the Declaration of Helsinki, and the protocol was approved by the research ethics committee of the College of Nursing Art and Science, University of Hyogo (KYOUIN24) on 12 March 2013. We invited the directors of the nursing care stations to participate in our study and obtained their consent. Subsequently, through them, we provided our letters of request to the residents and their families. We verbally explained our study using the explanatory documents. All participants provided their informed consent for inclusion in the study.

## 3. Results

### 3.1. Subjects’ Characteristics

The demographic data of the subjects are shown in Table 1. A total of 227 subjects (mean age, 86.2 ± 8.0 years; 78.9% female) participated in this study. The gender composition in this study (Table 1) was similar to the data of the national survey of Japan (male, 22.6%; female, 77.4%) [16]. All data were combined for further analysis because no particular different tendencies in gender were observed in this study. Overall, the degrees of independence in performing ADLs ranged from “remains independent if any person attends to him/her” to “frequently shows difficulty in performing ADLs and always requires nursing care”. The comorbid illnesses included dementia in 122 (53.7%) participants, past history of stroke in 73 (32.2%) participants, and hypertension in 70 (30.8%) participants.

### 3.2. Relationship between the Consistency of Food and Pneumonia/Fever

The consistencies of foods were categorized as regular, soft-solid, chopped, paste, and gastric tube foods (Table 1).

Table 2 summarizes the relationship between the consistencies of foods and past history of pneumonia as well as the presence/absence of fever over the past three months. A significantly higher number of participants with past history of pneumonia ate foods without chewing, i.e., they consumed paste and gastric tube foods, than did those without past history of pneumonia (*p* < 0.001). Based on the results of the relationship between the consistencies of foods and episodes of fever in the past three months, participants who consumed food without chewing, i.e., paste and gastric tube foods, suffered significantly more from fever than did those who chewed their food (*p* < 0.001).

### 3.3. Relationship between the Consistencies of Food and BMI, Calorie Intake, Water Intake, and Appetite

Table 2 summarizes the data regarding BMI, calorie intake, and water intake according to the consistencies of food. Significant differences were observed in the results of one-way analysis of variance. The participants who received regular food had significantly higher BMI than did those who received other consistencies of foods; furthermore, their mean calorie intake was significantly higher than those who received chopped, paste, or liquid foods via the gastric tube. Similarly, their water intake was also significantly higher than those who did not receive regular food.

Table 3 summarizes the results of the relationship between the consistencies of food and appetite. The closer the food was to regular food in terms of consistency, the greater were the number of participants who had an appetite (*p* < 0.001). Table 3 does not include “gastric tube feeding”, because these participants could not eat by mouth for ≥1 year because of difficulty in swallowing due to cerebral infarction or old age.

### 3.4. Relationship between the Consistencies of Food and Choking and the Conditions of Oral Cavity

The frequency of choking was low among the participants who received regular food; however, it was high among those who received chopped or paste foods (*p* < 0.001; Table 3).

Subjects who consumed regular food had a higher incidence of good dental occlusions in comparison to those who did not receive regular food, whereas the incidence of faulty occlusion was higher among those who received chopped or paste food (*p* < 0.001; Table 3). Additionally, a greater number of participants who received regular food had good tongue movement and control, while those who received chopped or paste food had poorer tongue movement and control (*p* < 0.001; Table 3).

### 3.5. Factors Involved in the Ingestion of Regular Food

Univariate logistic regression analysis was performed to determine whether regular food was received, and the following results were obtained. Age was not significantly correlated, whereas good dental occlusions, the number of residual teeth, tongue movement and control, appetite, VI, and BMI were positively correlated (*p* < 0.000–0.05) with the consumption of regular food. Level of care needed, daily life degree of autonomy, past history of pneumonia, frequency of fever, and frequency of choking were negatively correlated with regular food intake (Table 4).

In the cases of the highly correlated predictors with each other, between VI and level of care needed, VI was selected; between tongue movement and control, tongue movement was selected; and between occlusal conditions and the number of residual teeth, the number of residual teeth was selected. Furthermore, past history of pneumonia, frequency of fever, appetite, and frequency of choking were also put into the multiple regression analysis. BMI was excluded because it reflected the consumption of regular food. The variables remaining in the final model were VI, appetite, number of residual teeth, and frequency of choking, and the discrimination predictive value was 84.4% (Table 5).

VI, appetite, frequency of fever, frequency of choking, number of residual teeth, and tongue movement were put into the analysis. Finally, the discrimination predictive value of 4 items was 84.4%.

## 4. Discussion

### 4.1. Importance of Regular Food

It has been reported that promoting oral intake results in better health because gastrostomy or tube feeding may result in nutritional deficiencies, which may result in an increased risk of pneumonia [17,18,19]. However, the researchers have directed their attention to the physical properties of food—such as consistency—for orally consumed food, mainly for the sake of safety. The present study clarified the following points. (i) The consistencies of foods were related to the episodes of pneumonia and fever, and fewer subjects who consumed regular food had both a past history of pneumonia and risk of fever than those who did not. (ii) The consistencies of foods were related to nutritional intake, and the food that resulted in the highest mean calorie intake was regular food, followed by chopped, paste, and gastrostomy tube foods. (iii) The consistencies of foods and choking were significantly related, and regular food, followed by chopped and paste foods, resulted in the least frequencies of choking. The consistency is modified based on an older person’s choking experiences during eating to allow for easier swallowing and prevent aspiration pneumonia. The incidence of choking was higher among the subjects who consumed food that did not require chewing in this study; therefore, whether modifications of the physical properties of foods resulted in any benefits remains unknown. (iv) Factors that enabled the intake of regular food, such as vitality, appetite, and the number of residual teeth, were also examined. From the comprehensive viewpoint, the findings of this study suggested that disease-related paralysis, reduced cognitive function, and aggravated oral conditions adversely affected the required consistencies of foods and nutritional conditions, and induced immune suppression, often leading to infections. In this vicious cycle, the health of such participants probably deteriorates over time.

Regular food requires chewing; therefore, it is expected to control dental diseases by promoting salivary secretions, improving oral hygiene, and maintaining immunity [10,11]. Furthermore, the use of masticatory organs and related muscles is expected to prevent disuse atrophy and maintain or enhance chewing and swallowing functions [20]. Regular foods have been reported to contribute toward the maintenance of good nutrition [21] because the calorie content in regular food per unit volume is higher than that in paste food. It has been reported that the colors, consistencies, tastes, and textures of regular foods could result in more satisfaction and mental stability [22,23] and that residents in nursing care facilities became perplexed upon seeing modified foods, which resulted in decreased food intake [24]. Mastication has been reported to enhance brain functions in humans [25] as well as animals [26,27], although further scientific evidence is needed. Chewing action activates tongue movement, which promotes the formation of food bolus and preparation for smooth swallowing [28]. The findings of this study also suggest some of these advantages of regular food.

### 4.2. Selection of Consistencies of Food

The following four levels of semisolid/solid foods were proposed by the US National Dysphagia Diet: (a) homogenous, very cohesive, pudding-like food, requiring very little chewing ability; (b) cohesive, moist, semisolid food, requiring some chewing ability; (c) soft food, requiring more chewing ability; and (d) regular food (all food allowed). Various Japanese authorities have established the standards such as (a) food requiring no chewing, (b) food that can be crushed by the tongue, (c) food that can be crushed by the gums, and (d) food that can be chewed easily. Chopped or paste food is not easy for the elderly to make a bolus of, and they are highly at risk of accidental swallowing, which is likely to remain in the oral cavity. The calorie content of pureed food per unit volume is low because of the amount of water added during the preparation. Furthermore, pureed food hardly appeals to the eyes and tastes of older people. Therefore, “soft food” has been introduced as an alternative consistency of food that can be chewed, facilitating bolus formation and swallowing, and is similar to regular food in appearance and taste. However, the efficacy of soft food has not been sufficiently demonstrated, and the high costs associated with its preparation remain problems to be solved.

Ideally, the consistency of food should be determined after assessing the chewing and swallowing abilities of older individuals; however, accurate assessment is not currently feasible. According to a study that investigated the food served to older nursing home residents, 91% of the residents received food with the physical properties far below their chewing ability, and only 5% of them received food with adequate physical properties [29]. Efforts should be made to improve chewing and swallowing functions among residents, with careful consideration of their health status.

### 4.3. Support for Dining

Logistic regression analysis in this study demonstrated that the number of residual teeth, appetite, and VI had a strong influence on regular food intake, suggesting the importance of willingness. Willingness has been regarded as a significant factor determining the prognosis in life [12]. In senior welfare facilities, the staff members should examine how they can provide nursing care that enhances willingness among residents.

The process of eating consists of the following stages: recognizing the food (preceding stage), chewing of food to form a bolus (preparatory stage), moving the food to the pharynx through the back of the tongue (oral stage), and, finally, swallowing [30]. Patients with Alzheimer’s disease gradually demonstrate difficulty in eating food by themselves because of marked damages that affect the preceding, preparatory, and oral stages of the eating process [31]. In the early stages of dementia, recognizing each patient as an individual in the interpersonal or social context is important [32].

In the preparatory stage, the teeth and tongue, which are used to chew food and form a bolus, and the relevant muscles should function in a coordinated manner. In this study, half of the subjects had faulty occlusions, resulting in difficulty in chewing food because of the limited number of teeth and high frequency of caries and ill-fitting dentures. In order to maintain chewing ability, the promotion of regular examinations as well as dental care for the prevention of dental caries and periodontal diseases should be emphasized before conditions requiring serious treatment arise.

### 4.4. The Clinical Implications of the Present Study

The findings of this study suggest that efforts should be made to improve the chewing and swallowing functions of residents with careful consideration of their health status since the consistency of food substances of regular food positively influences the health of elderly individuals.

### 4.5. Limitation of the Present Study and Future Issues

This was a cross-sectional study, which could not establish causal relationships and could only suggest the presence of a relationship. In the future, we intend to perform an interventional study on this topic and promote the serving of food that is as close to regular food as possible in terms of consistency. Additionally, we need to evaluate the results of this study and examine the influence of declines in the consistency of food on the health of older people.

## 5. Conclusions

Regular consistency of food tended to be associated with a low frequency of pneumonia and fever, high-calorie intake, and a low incidence of choking. Factors that enabled the eating of regular food were the presence of a higher vitality index, appetite, and the number of teeth and good occlusal conditions.

## Figures and Tables

**Table 1:** Feature of subjects.

**Gender**	**N**	**%**	
Male	48	21.1	
Female	179	78.9	
Variables	Mean	SD	Range
Age	86.2	8.0	60–104
BMI	19.6	3.7	10.8–36.0
**Consistency of Food Substances**	**N**	**%**	
Regular	48	21.1	
Soft-solid	36	15.9	
Chopped	78	34.4	
Paste	37	16.3	
Gastric-tube	28	12.3	

**Table 2:** Consistency of food substances and physical condition.

		1. Regular	2. Soft-Solid	3. Chopped	4. Paste	5. Gastric Tube	*p*-Value	Post Hoc ^(3)^
Variables		N = 48	N = 36	N = 78	N = 37	N = 28		
History of pneumonia	none	40 (83.3%)	26 (72.2%)	55 (70.5%)	14 (37.8%)	10 (35.7%)	<0.001 ^(1)^	
	exist	8 (16.7%)	10 (27.8%)	23 (29.5%)	23 (62.2%)	18 (64.3%)		
Fever within 3 months	none	41 (85.4%)	29 (80.6%)	58 (74.4%)	19 (51.4%)	10 (35.7%)	<0.001 ^(1)^	
	exist	7 (14.6%)	7 (19.4%)	20 (25.6%)	18 (48.6%)	18 (64.3%)		
BMI	mean	21.9	20.3	19.5	17.7	17.3	<0.001 ^(2)^	1 > 2, 3 > 4 > 5
	S.D.	4.3	3.2	3.3	1.9	3.5		
Calorie intake	mean	1325.9	1318.7	1125.0	1122.0	812.5	<0.001 ^(2)^	1, 2 > 3, 4 > 5
	S.D.	220.2	235.3	256.8	288.5	150.7		
Water intake	mean	1292.5	1198.5	1116.7	1053.5	533.2	<0.001 ^(2)^	1, 2 > 2, 3, 4 > 5
	S.D.	321.7	351.5	347.9	272.9	174.7		

^(1)^ Results of χ^2^ test between categories of vertical items and 5 categories of food substances. ^(2)^ Results of ANOVA. ^(3)^ Results of post hoc comparisons (Tukey–Kramer method) *p* < 0.05 after one-way ANOVA.

**Table 3:** Consistency of food substances and appetite and oral condition.

Variables		Regular	Soft-Solid	Chopped	Paste	*p*-Value ^(1)^
		N = 48	N = 36	N = 78	N = 37	
Appetite	good	27 (56.3%)	15 (41.7%)	25 (32.1%)	9 (24.3%)	<0.001
	normal	19 (39.6%)	15 (57.1%)	26 (33.3%)	20 (54.1%)	
	none	2 (4.2%)	6 (16.7%)	27 (34.6%)	8 (21.6%)	
Frequency of choking	none	41 (85.4%)	25 (69.4%)	40 (51.3%)	12 (32.4%)	<0.001
	several times/day	5 (10.4%)	10 (27.8%)	26 (33.3%)	15 (40.5%)	
	every meal time	2 (4.2%)	1 (2.8%)	12 (15.4%)	10 (27.0%)	
Dental occlusions	good	33 (67.3%)	14 (38.9%)	28 (36.4%)	9 (24.3%)	<0.001
	bad	16 (32.7%)	22 (61.1%)	49 (63.6%)	28 (75.7%)	
Tongue movement	0	4 (8.3%)	7 (19.4%)	22 (28.2%)	19 (51.4%)	<0.001
	1	5 (10.4%)	1 (2.8%)	16 (20.5%)	4 (10.8%)	
	2	11 (22.9%)	10 (27.8%)	11 (14.1%)	4 (10.8%)	
	3	28 (58.3%)	18 (50.0%)	29 (37.2%)	10 (27.0%)	
Tongue control	0	4 (8.3%)	7 (19.4%)	22 (28.2%)	21 (56.8%)	<0.001
	1	5 (10.4%)	2 (5.6%)	17 (21.8%)	7 (18.9%)	
	2	39 (81.3%)	27 (75.0%)	39 (50.0%)	9 (24.3%)	

^(1)^ Results of χ^2^ test between categories of vertical items and 4 categories of food substances.

**Table 4:** Univariate logistic regression analysis.

Variables	Regression Coefficient	OR	95% CI	*p*-Value
Age	−0.002	0.998	(0.958, 1.039)	n.s.
BMI	0.209	1.233	(1.117, 1.361)	<0.001
Level of care needed	−0.830	0.436	(0.289, 0.658)	<0.001
Daily life degree of autonomy	−0.854	0.436	(0.240, 0.755)	<0.01
History of pneumonia	−0.945	0.389	(0.175, 0.861)	<0.05
Fever within 3 months	−0.944	0.389	(0.162, 0.931)	<0.05
Appetite	1.282	3.606	(2.046, 6.323)	<0.001
Frequency of choking	−1.298	0.273	(0.136, 0.547)	<0.001
Vitality Index	0.545	1.725	(1.431, 2.079)	<0.001
Occlusal condition	1.387	4.004	(2.016, 7.950)	<0.001
Number of residual teeth	0.089	1.093	(1.050, 1.137)	<0.001
Tongue movement	0.615	1.849	(1.078, 3.171)	<0.05
Tongue control	1.394	4.032	(1.384, 11.746)	<0.05

**Table 5:** Logistic regression analysis.

Variables	Regression Coefficient	SE	Wald	OR	95% CI	*p*-Value
Vitality Index	0.353	0.092	14.802	1.424	(1.189, 1.704)	<0.001
Appetite	0.634	0.311	4.165	1.886	(1.025, 3.468)	<0.05
Number of residual teeth	0.092	0.027	11.536	1.097	(1.040, 1.156)	<0.001
Frequency of choking	−0.949	0.398	5.690	0.387	(0.178, 0.844)	<0.05
Constant	−6.418	1.385	21.490			
